# Dressler’s Syndrome as a Late Complication of Myocardial Infarction: A Case Report

**DOI:** 10.7759/cureus.69583

**Published:** 2024-09-17

**Authors:** Mateusz Wilk, Krzysztof Patela, Dominik Krupka, Jakub Ptak, Agata Malczyk

**Affiliations:** 1 Department of Cardiology, Marciniak Lower Silesian Specialist Hospital, Wrocław, POL; 2 Institute of Internal Medicine - Department of Diabetology, Hypertension and Internal Medicine, University Hospital of Wrocław, Wrocław, POL; 3 Institute of Heart Diseases - Student Scientific Club of Transplantology and Advanced Therapies of Heart Failure, Wrocław Medical University, Wrocław, POL; 4 Institute of Heart Diseases, University Hospital of Wrocław, Wrocław, POL

**Keywords:** complication of myocardial infarction, dressler's syndrome, internal diseases, pericarditis, post-cardiac injury syndrome

## Abstract

Dressler's syndrome, a rare complication of myocardial infarction, is a form of secondary pericarditis that typically develops within one to six weeks following the infarction. In this report, we present a case of a 68-year-old woman who was diagnosed with Dressler’s syndrome after nine weeks from acute coronary syndrome. The patient first presented with an ill-tolerated episode of atrial fibrillation. Based on clinical, laboratory, and imaging findings, accompanied by a history of respiratory tract infections, an infection of an unknown origin was initially suspected. Lack of response to the applied treatment prompted the suspicion of Dressler's syndrome. This was followed by a cardiac MRI, which showed features of pericarditis. The patient responded well to a seven-day treatment with ibuprofen and was discharged home in good general condition.

## Introduction

Dressler’s syndrome is a form of secondary pericarditis that occurs following an injury to the heart, typically a myocardial infarction (MI). It belongs to a broader group of conditions collectively called post-cardiac injury syndromes (PCIS) [1]. While the pathophysiology of Dressler’s syndrome is not fully understood, it is believed it originates from an autoimmune mechanism initiated by a release of myocardial antigens during the primary injury to the heart [1-3].

It typically occurs within the first two weeks after the MI [4], although the latency period is highly variable [3]. Usual symptoms include generalized weakness, pleuritic chest pain, fever, dyspnea, and palpitations [1]. Pleural and/or pericardial effusion along with elevated inflammatory markers, such as C-reactive protein (CRP) and erythrocyte sedimentation ratio, are common findings [1,2]. While an electrocardiogram (ECG) is usually abnormal, the classical signs of pericarditis, such as widespread ST-segment elevations and PR-segment depressions, are not often seen [1]. The clinical course is usually benign, and the disease responds well to treatment with anti-inflammatory drugs, including non-steroidal anti-inflammatory drugs (NSAIDs) [1-3].

Once more prevalent, Dressler's syndrome is now a rare condition, occurring in less than one percent of all MI patients [5,6]. This is believed to be due to advances in the treatment of acute coronary syndromes (ACS) [6,7]. We discuss the case of a 68-year-old Caucasian woman in whom Dressler’s syndrome occurred as a late complication, with the first symptoms presenting after nine weeks from MI.

## Case presentation

A 68-year-old woman with hypertension and paroxysmal atrial fibrillation (AF) was admitted to the emergency department due to an episode of AF. She complained of palpitations and decreased exercise tolerance.

Nine weeks earlier, she had suffered an ST-segment elevation MI (STEMI). Initial symptoms of chest pain began five days before she presented to the hospital. After the admission to the hospital, an immediate angioplasty with implantation of two drug-eluting stents (DES) within the right coronary artery was performed. In addition, due to stenosis in the left anterior descending artery detected at the coronary angiography, the patient was qualified for a second-stage angioplasty, which was conducted during the same hospitalization. Implantation of DES and drug-eluting balloon angioplasty were performed. She was discharged with the recommendation of starting a cardiac rehabilitation program.

Subsequently, she was hospitalized in the cardiac rehabilitation unit of a different hospital, where she was observed to have an increase in CRP, symptoms of a respiratory tract infection, and bilateral pleural effusion. The patient was transferred to the pulmonary disease unit, where bronchitis was diagnosed. Applied anti-inflammatory treatment resulted in the resolution of symptoms and a decrease in inflammatory parameters.

On admission, the patient presented with irregular rhythm and an increased heart rate of 120 beats per minute, blood pressure of 115/70 mm Hg, and body temperature of 36.7 °C. A physical exam was significant for basal crackles over the left lung on auscultation and slight edema around both ankles.

The ECG showed AF with ventricular action around 120 beats per minute. Elevated inflammatory parameters and N-terminal prohormone of brain natriuretic peptide (NT-proBNP) concentration were observed (Table 1). Cardiac troponin T was negative. Blood and urine samples were obtained for culture. A transthoracic echocardiogram revealed a circumferential pericardial effusion, grade II diastolic dysfunction, impaired right ventricular function, and a slightly decreased left ventricular ejection fraction.

**Table 1:** Key admission parameters CRP: C-reactive protein, NT-proBNP: N-terminal prohormone of brain natriuretic peptide

Parameter	Value	Reference range
White blood count	12,000/µl	4,000-10,000/µl
CRP	214 mg/l	0-5 mg/l
NT-proBNP	4364 pg/ml	0-125 pg/ml

**Figure 1:**
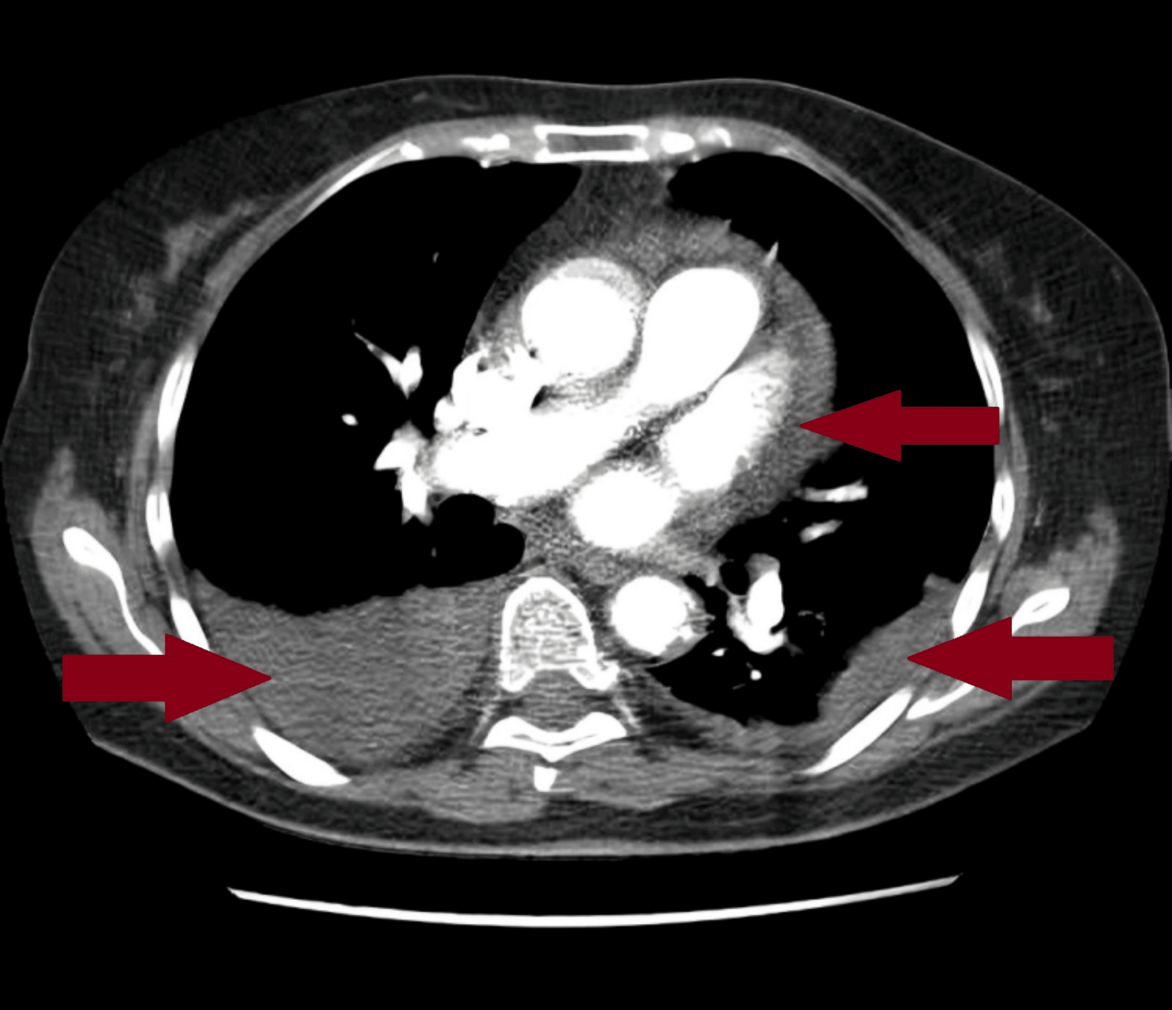
Admission CT A pericardial effusion 10 mm in diameter, a left-sided pleural effusion 15 mm in diameter, and a right-sided pleural effusion 38 mm in diameter are seen. CT: computed tomography

CT of the thorax showed a pericardial effusion 10 mm in diameter, a left-sided pleural effusion 15 mm in diameter, and a right-sided pleural effusion 38 mm in diameter and bibasilar atelectasis (Figure 1).

Based on the history of respiratory tract infections and clinical, laboratory, and imaging findings, an infection of unknown origin that triggered an episode of AF was suspected. Subsequently, antibiotic therapy with ceftriaxone and levofloxacin was initiated. In the following days, Lyme disease and tuberculosis were ruled out based on antibody assays, and the levels of numerous autoantibodies were determined. Assays of rheumatoid factor, antimitochondrial antibodies M2 (AMA-M20), anti-gp210, anti-PGDH, anti-PML, and anti-sp100 were positive, but no unequivocal diagnosis of autoimmune disease was established.

**Figure 2:**
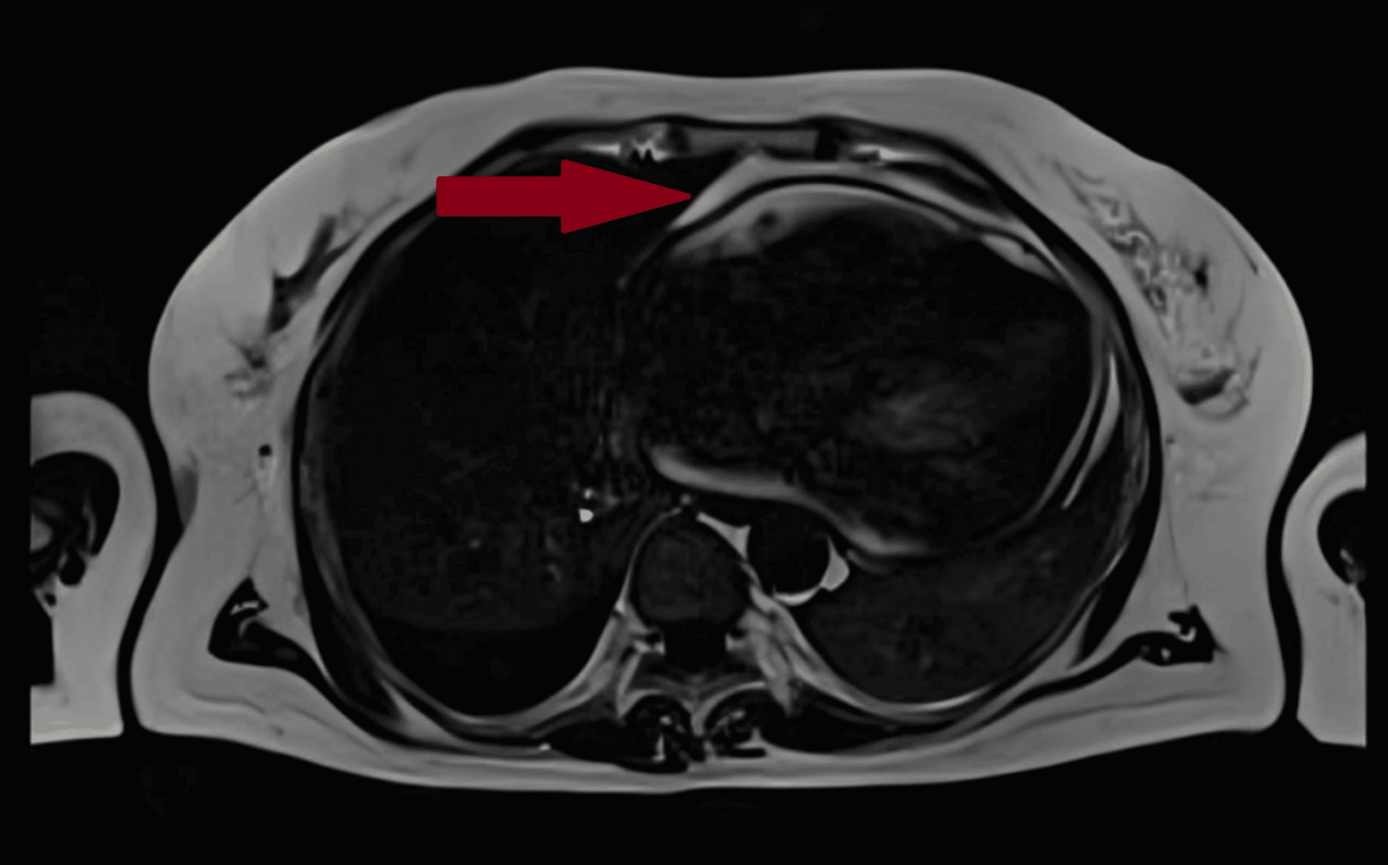
Cardiac MRI T1-weighted sequence. Thickening of the pericardium is seen. MRI: magnetic resonance imaging

In parallel, Dressler's syndrome was suspected due to the history of STEMI, persistent malaise, constantly elevated inflammatory parameters, negative blood and urine cultures, and no other clear diagnosis. An MRI of the heart was performed. It showed thickening of the pericardium up to 6 mm in the inferior and lateral wall regions, clearly visible in the T1-weighted sequence (Figure 2). The image was consistent with acute pericarditis, which confirmed the diagnosis of Dressler’s syndrome. Consequently, ibuprofen was included in the treatment. Due to ongoing antiplatelet therapy with clopidogrel after ACS, a proton pump inhibitor was added.

**Figure 3:**
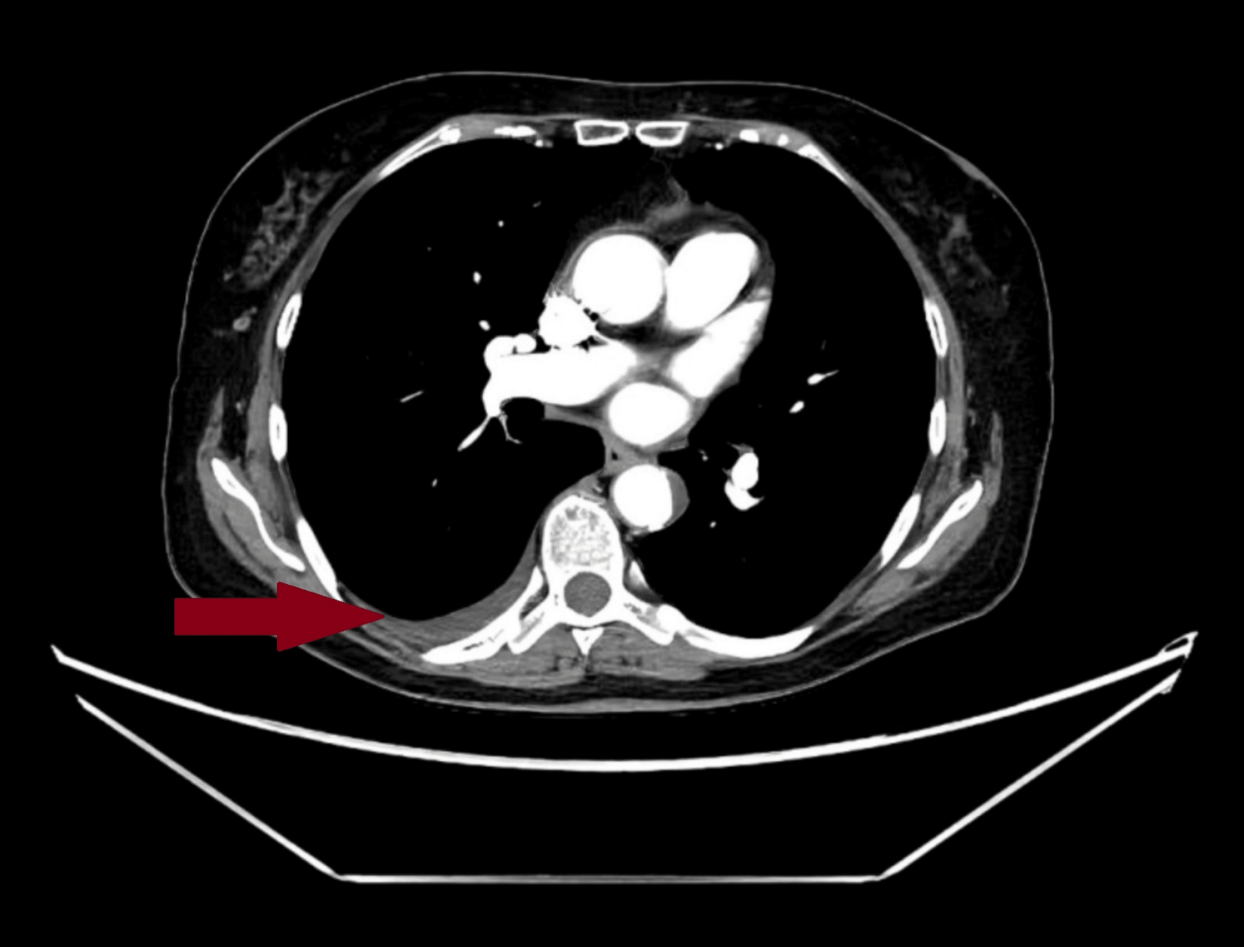
CT at discharge Visible resolution of pericardial effusion and fluid in the left pleural cavity. 7 mm of fluid in the right pleural cavity is seen. CT: computed tomography

After seven days of ibuprofen treatment, the patient's condition significantly improved. Inflammatory markers decreased, and repeat CT showed resolution of the pericardial effusion along with a significant reduction in the volume of fluid in both pleural cavities (Figure 3). The patient was discharged in good condition (CRP concentration of 5,58 mg/l and white blood count of 5,670/µl) with the recommendation of attending a rheumatology consultation.

## Discussion

Our case report presents a 68-year-old woman in whom Dressler’s syndrome presented as a late complication of MI. We believe that this case carries a message of relevance for professionals caring for patients after ACS, highlighting the importance of several elements considered in the pathogenesis of the condition.

Dressler’s syndrome was first described in 1956. Before reperfusion therapies, that is, thrombolysis and coronary balloon angioplasty were widely implemented, it had been estimated that it would occur in about one to five percent of all MI patients [7]. Currently, it is a rare complication, affecting less than one percent of all MI patients [5,6].

The decrease in its prevalence is attributed to reperfusion therapies, which limit the damage to the myocardium and, therefore, decrease the release of myocardial antigens [6,7]. Exposed antigens are believed to play a crucial role in the pathogenesis of Dressler’s syndrome, triggering an autoimmune process that leads to pericarditis [1-3,8]. This is consistent with observations that the current few cases of the syndrome were associated with massive myocardial damage [6]. It was proposed that viruses may also contribute to the pathogenesis, as seasonal variation was reported and elevated viral titers were associated with the syndrome [9].

In our case, the patient presented to the hospital and underwent PCI after five days from the onset of symptoms of MI. Delay in reperfusion probably led to an extensive release of myocardial antigens and increased risk of starting the autoimmune cascade. Secondly, the patient probably had bronchitis a few weeks before the described hospitalization, underscoring the postulated role of viruses in the pathogenesis of the disease. On the other hand, it is also possible that the symptoms first interpreted as bronchitis were in fact due to Dressler's syndrome. It would then be appropriate to interpret this case as a recurrence of Dressler's syndrome after anti-inflammatory treatment targeted at bronchitis relieved its symptoms. Recurrence of PCIS is not uncommon [1].

Dressler’s syndrome typically occurs within the first two weeks after the MI [4], which is significantly shorter than the nine weeks that we observed. However, the latency varies, and it can be diagnosed later [3]. Most common signs and symptoms, such as malaise, pleuritic chest pain, low-grade fever, and elevated inflammatory markers, are non-specific [1]. Classic ECG findings of diffuse ST-segment elevations and PR depressions are rare [1]. The suspicion of the diagnosis can be made based on the findings of pleural and pericardial effusion, which are present in most patients [1]. However, Dressler’s syndrome is currently a rare condition, so other possible diagnoses are more likely to be considered first. Further causes of pericarditis include tuberculosis, which should be considered especially in endemic regions, other bacterial and viral infections, autoimmune diseases, cancer, and uremia [10].

## Conclusions

Although Dressler's syndrome is now a rare complication, it should always be considered in patients after ACS with persistently elevated inflammatory parameters and other often non-specific symptoms such as palpitations or weakness. A history of vast myocardial damage and viral infections should further increase the physician’s alertness. It should not be forgotten that recent treatment with commonly used NSAIDs can blur the clinical picture, additionally complicating the diagnosis of an already uncommon condition.
